# Pharmacokinetic and pharmacodynamic strategies to optimize antifungal therapy, prevent resistance and minimize treatment failure

**DOI:** 10.1093/jacamr/dlag231

**Published:** 2026-11-04

**Authors:** Norman Van Rhijn, P Lewis White, Riina Rautemaa-Richardson

**Affiliations:** Department of Biology, University of Oxford, Oxford, UK; Public Health Wales (PHW) Mycology Reference Laboratory, University Hospital of Wales, Cardiff, UK; Division of Infection and Immunity, School of Medicine, Cardiff University, Cardiff, UK; Mycology Reference Centre Manchester, ECMM Centre of Excellence, and Department of Infectious Diseases, Manchester Academic Health Science Centre, Wythenshawe Hospital, Manchester University NHS Foundation Trust, Manchester, UK; Division of Evolution, Infection and Genomics, Faculty of Biology, Medicine and Health, University of Manchester, Manchester, UK

## Abstract

Invasive fungal diseases (IFDs) present significant challenges in clinical practice due to high morbidity and mortality rates, compounded by antifungal resistance, diagnostic limitations and pharmacokinetic (PK) and pharmacodynamic (PD) complexities. The main antifungal classes, triazoles, polyenes, echinocandins and pyrimidine analogues, are essential for managing IFDs but are increasingly undermined by resistance due to genetic mutations, efflux pump over-expression and biofilm formation. Some fungal pathogens, such as *Candidozyma auris*, can resist multiple antifungal classes by deploying a combination of mechanisms. PK/PD indices, including *C*_max_/minimum inhibitory concentration (MIC), AUC/MIC and percentage of time (%*T*) > MIC, are essential for optimizing antifungal therapy and improving the likelihood of adequate efficacy, while potentially limiting resistance selection through improved exposure. However, achieving these targets is often hindered by factors such as physiological variability, drug–drug interactions and inadequate tissue penetration, particularly in difficult-to-treat infections like fungal meningitis. Newer antifungal agents, including rezafungin, with its once-weekly dosing, and ibrexafungerp, the first-in-class oral triterpenoid, offer potentially useful additions to current therapy, although clinical experience and resistance-related outcome data remain limited for newer agents. These drugs feature enhanced PK/PD profiles and activity against resistant strains. Furthermore, therapeutic drug monitoring and advanced modelling techniques, such as Monte Carlo simulations, are vital for tailoring treatment regimens to individual patients. This review highlights the importance of integrating PK/PD principles, novel antifungal therapies and stewardship strategies to optimize antifungal exposure and support patient management in IFDs. These approaches may help reduce treatment failure and resistance selection, although direct clinical evidence for prevention of acquired resistance remains limited.

## Introduction

Invasive fungal diseases (IFDs) present a significant challenge in clinical practice, requiring effective antifungal treatment strategies to combat high morbidity and mortality rates.^1^ However, rapid and effective treatment is frequently hindered by limited diagnostic availability, increasing antifungal resistance and pharmacokinetic (PK) and pharmacodynamic (PD) complexities.

Antifungal agents approved to treat IFDs are categorized into four main classes: triazoles, polyenes, echinocandins and pyrimidine analogues. Each class targets distinct fungal structures or processes, such as disrupting nucleic acid synthesis, ergosterol biosynthesis, cell membrane integrity or cell wall synthesis.^2,3^ While these agents are critical for managing IFDs, their clinical utility is increasingly compromised by resistance due to genetic changes and by tolerance due to biofilm formation, which reduce drug efficacy and limit treatment options.^4–13^ The rise of antifungal resistance, particularly in yeasts, is associated with the wide clinical use of antifungals, and in moulds, is primarily driven by environmental pressures caused by widespread use of agricultural azole fungicides and subsequent cross-resistance with medical antifungals.^14^ Resistance mechanisms, including mutations in various genes (e.g. *ERG11/CYP51*, *FKS*), gene over-expression, as well as chromosomal aneuploidy, enable fungal pathogens to overcome the effects of antifungal agents.^7,8,15^ Notably in fungi, resistance often comes with minimal fitness costs, allowing fungal pathogens to retain their virulence and adaptability.^16^ Mutator isolates can further accelerate resistance development by harbouring defects in DNA repair pathways that enhance mutation rates under selective pressure.^17^ Compounding these challenges, multidrug-resistant species, such as *Candidozyma auris*, have emerged as a global healthcare threat due to their potential resistance to multiple antifungal classes (azoles, echinocandins and polyenes), high transmissibility and ability to form robust biofilms that persist on healthcare surfaces, fuelling nosocomial outbreaks.^18^ This complex resistance landscape requires improved treatment approaches, combined with the integration of rapid effective diagnostics and robust stewardship efforts.^2,19,20^ In this context, antifungal stewardship includes not only avoiding unnecessary therapy but also ensuring the right drug, dose, formulation, route and duration are used for the infecting organism, site of infection and patient-specific PK/PD factors, thereby helping to minimize both treatment failure and avoidable resistance selection.

PK and PD principles play a pivotal role in optimizing antifungal therapy and limiting resistance.^21,22^ PK/PD indices, including the percentage of time above the minimum inhibitory concentration (%*T*/MIC), the area under the curve to the MIC (AUC/MIC) and the maximum concentration to the MIC (*C*_max_/MIC), are essential for guiding dosing strategies and achieving therapeutic success, and are well described in the literature.^22,23^ However, achieving these targets in clinical practice is challenging due to factors such as physiological variability, drug–drug interactions (DDIs) and inadequate tissue penetration, especially for difficult-to-treat infections like fungal meningitis. In this review, subtherapeutic exposure refers to drug concentrations insufficient to achieve the relevant PK/PD target (PDT) at the site of infection in relation to the organism MIC. By itself, subtherapeutic exposure demonstrates only inadequate exposure and does not prove resistance; where it results in an inability to control the infection, we describe this as persistent infection or clinical failure (a lack of microbiological or clinical response), which is distinct from resistance. Antifungal resistance refers to a heritable reduction in susceptibility, typically demonstrated by elevated MICs and/or defined resistance mechanisms. Throughout this review we reserve ‘resistance’ for such evidence; where studies show only inadequate exposure, persistent infection or clinical failure, we describe them as such. Where evidence supports it, prolonged inadequate exposure may also select for less-susceptible or resistant subpopulations.

Unlike existing reviews that address antifungal PK/PD, tissue penetration and resistance separately, this review integrates these areas into a single exposure-based framework linking drug concentration, pathogen susceptibility and infection site to clinical outcome. We connect resistance mechanisms to inadequate drug exposure and treatment failure, extend the discussion to newer, approved antifungal agents and provide therapeutic drug monitoring (TDM)-informed dosing guidance for special patient populations, with the aim of supporting clinicians in delivering effective, individualized antifungal therapy. Ultimately, understanding and applying PK/PD principles are critical to optimizing antifungal exposure and minimizing treatment failure in the management of IFDs, while their direct effect on preventing acquired resistance in clinical practice remains less well established.

## Methods

This narrative review was informed by a PubMed search conducted in 2025, covering literature from January 2000 onwards, with earlier work included where foundational. Search terms included antifungal PK/PD, TDM, resistance and treatment failure, major drug classes and key concepts including MIC, mutant selection window (MSW), Monte Carlo simulation, biofilm and IFD; newer antifungal agents were also searched by name. A draft outline based on the initial literature search was discussed at an author roundtable on 20 March 2026, where experts suggested additional papers and shaped the review’s scope, followed by several rounds of revision. Notably, this review focuses on antifungal agents that are currently approved and in clinical use.

As this is a narrative review, evidence was prioritized pragmatically rather than through a formal systematic process. The review addresses mechanistic and exposure-based questions that are often better answered by *in vitro* and animal PK/PD studies, pharmacometric modelling, observational clinical data and resistance-mechanism research than by randomized clinical trials (RCTs) alone. The evidence base therefore draws primarily on these sources, with RCTs included where comparative clinical efficacy data are available.

## Role of PK/PD indices, MIC and TDM in antifungal therapy management

Efficient antifungal therapy hinges on adequately linking PK, PD and MIC to guide drug dosage and administration strategies (Figure 1).^21,22^ A PK/PD index is the measure that most directly correlates drug exposure with efficacy. The three established PK/PD indices, *C*_max_/MIC, AUC/MIC and %*T*/MIC, describe the relationship between dose, pathogen susceptibility and therapeutic effect, as previously reviewed.^22,23^  *C*_max_/MIC and AUC/MIC are the primary indices for antifungal drugs with concentration-dependent activity, where increased drug exposure enhances efficacy (Table 1).^22^ For drugs whose PDT is defined by *C*_max_/MIC or AUC/MIC, the rate and extent of pathogen killing generally increases as drug concentration rises. Efficacy depends on achieving concentrations above the MIC of the target pathogen for the specific therapy.^22^ Therefore, such drugs achieve optimal efficacy when administered in large, infrequent doses. Treatment failure can occur if the pathogen already has a high MIC (i.e. is already resistant) to the therapy or if the drug concentration achieved in the patient is lower than predicted.^22^ Prolonged or repeated low exposure may also favour selection of less-susceptible subpopulations, although inadequate exposure more directly causes failure to achieve the desired therapeutic effect. Conversely, %*T* > MIC applies to time-dependent (or concentration-independent) drugs, where maintaining concentrations above the MIC for a defined duration is critical for efficacy.^22,53^ Time-dependent activity requires smaller, more frequent doses to ensure consistent therapeutic levels, but the same efficacy considerations remain.^22,54^ Each antifungal drug class aligns with a preferred PK/PD index that reflects its efficacy in clinical practice (Table 1; Figure 1). It is also important to consider how these indices are influenced by protein binding, as only the unbound (free) drug is pharmacologically active and subtherapeutic exposure may promote antifungal resistance.^55^ Highly bound agents such as itraconazole (>99.8% protein bound) and posaconazole (∼99% protein bound) may require higher total drug concentrations to achieve effective free drug levels that minimize the opportunity for resistance development.^56^ Protein-bound antifungal agents can be significantly sequestered during extracorporeal membrane oxygenation (ECMO; e.g. micafungin).^57^

**Figure 1.**
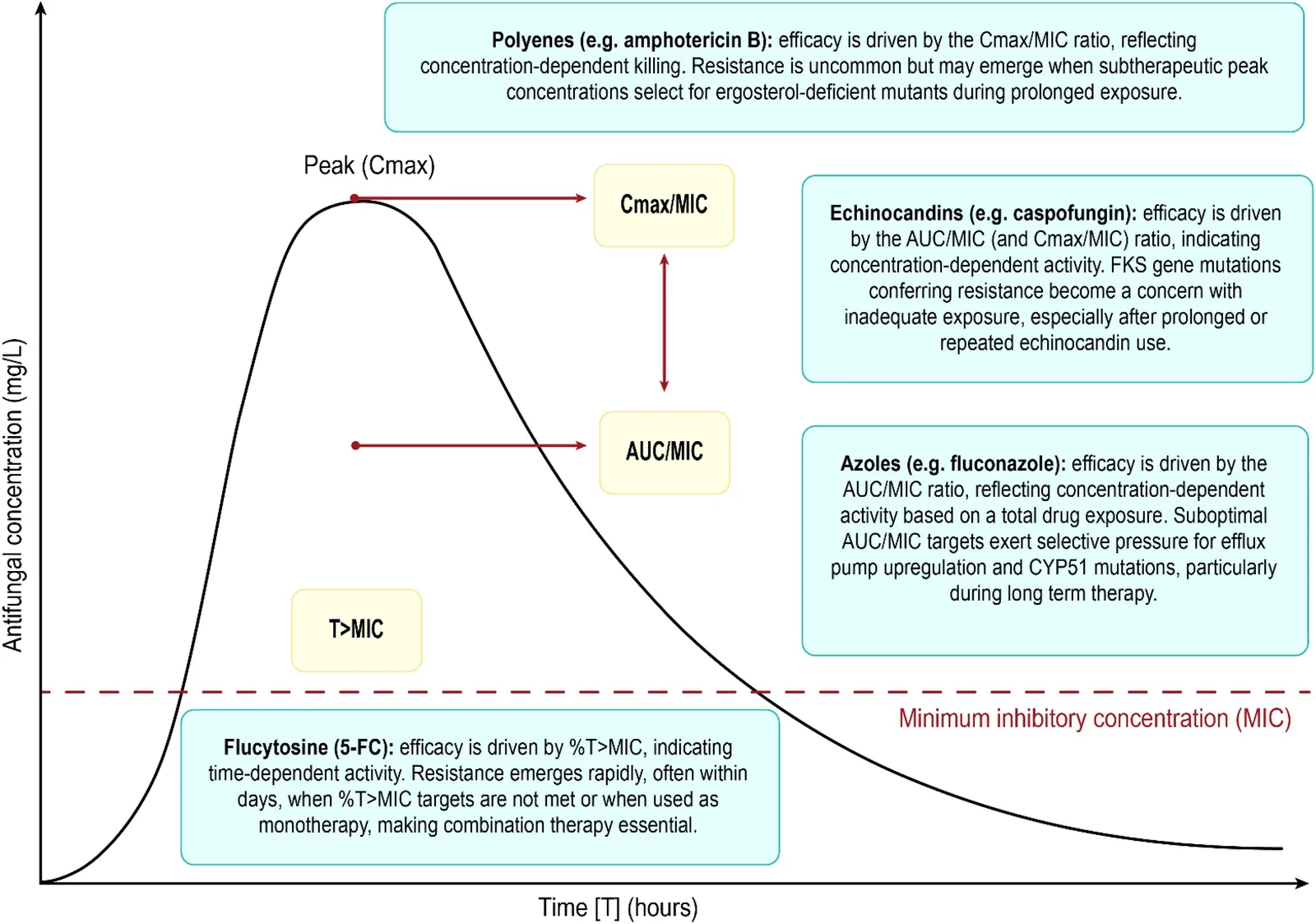
Schematic of PK/PD indices across commonly used antifungal classes and their implications for activity and resistance. 5-FC, flucytosine; AUC, area under the concentration–time curve; *C*_max_, maximum concentration; MIC, minimum inhibitory concentration; PK/PD, pharmacokinetic/pharmacodynamic; %*T* > MIC, percentage of time that drug concentrations remain above the MIC.

**Table 1.** PK/PD indices and thresholds by antifungal drug class

Antifungal name	PK/PD index	Example target values	Comments	References
*Triazoles* ^a^				
Voriconazole	AUC_0-24_/MIC	AUC_0-24_/MIC target of 11–58 for *C. albicans*; trough levels of 2–6 mg/L suggested for *Aspergillus* spp.	TDM is required due to narrow therapeutic index and variability in metabolism. Ensures efficacy and avoids toxicity.	^24–26^
Posaconazole	AUC_0-24_/MIC, *C*_min_	AUC_0-24_/MIC of 6.12–26.7 for *C. albicans*; *C*_min_ > 0.7 mg/L for prophylaxis; >1 mg/L for treatment	TDM needed to ensure adequate absorption and target trough levels, especially for prophylactic use.	^27–29^
Fluconazole	AUC_0-24_/MIC	AUC_0-24_/MIC of 400–800 for *Candida* spp. *Candido. auris* is almost universally resistant (MIC typically ≥32 mg/L), rendering standard AUC_0-24_/MIC targets unachievable and fluconazole unsuitable for treatment	Monitoring is unnecessary in most cases due to predictable PK but it is crucial in special populations (e.g. critical illness).	^30,31^
Isavuconazole	AUC_0-24_/MIC, *C*_min_	AUC_0-24_/MIC of 11.1 for *Aspergillus* spp.; suggested trough levels ≥1 mg/L for efficacy.	TDM may be necessary for complex cases, especially with interacting medications or in severe infections.	^32–34^
Itraconazole	AUC_0-24_/MIC, *C*_min_	AUC_0-24_/MIC of 25–50 for *Aspergillus* spp.; *C*_min_ >0.5–1 mg/L for efficacy	TDM is required due to unpredictable absorption and significant drug interactions. Achieving optimal levels is critical for efficacy.	^35,36^
*Polyenes*				
Amphotericin B	*C*_max_/MIC	*C*_max_/MIC ratio of 2.4 for *Aspergillus* spp.	High peak concentration is essential for efficacy. Lipid formulations mitigate toxicity while maintaining PK/PD goals.	^37–39^
Liposomal amphotericin B	*C*_max_/MIC	Target efficacy linked to *C*_max_/MIC >1 for *Candida* and *Aspergillus* spp. In vitro PK/PD modelling shows the *C*_max_/MIC for stasis is ∼9 for *Candido. auris*^b^	Liposome formulation improves drug delivery to infection sites, optimizing PK/PD and reducing nephrotoxicity.	^40,41^
*Echinocandins*				
Caspofungin	*C*_max_/MIC, AUC_0-24_/MIC	AUC_0-24_/MIC 865 for *C. albicans* and 450 for *N. glabrata*	PK/PDT ensures fungicidal activity against most *Candida* spp. Regular therapy monitoring confirms exposure consistency.	^42^
Anidulafungin	AUC_0-24_/MIC	AUC_0-24_/MIC target value of 20 for *C. albicans* and 7 for *N. glabrata*	Determining PK/PD levels is critical for efficacy and dose adjustment. Lack of hepatic metabolism minimizes variability.	^43^
Micafungin	AUC_0-24_/MIC	AUC_0-24_/MIC of 285 for *C. parapsilosis* and 5000 for *C. albicans* and *N. glabrata*	Monitoring key for optimizing efficacy against high MIC strains like *C. parapsilosis*.	^44^
Rezafungin	fAUC_0-168_/MIC	fAUC_0-168_/MIC targets for stasis across *Candida* spp.: 20.5 for *C. albicans*, 0.5 for *N. glabrata*, 18.2 for *C. parapsilosis* and 12.1 for *Candido. auris*^c^	A metabolically and chemically stable echinocandin, characterized by low Cl and a *t*_1/2_, enabling once-weekly dosing. As a concentration-dependent drug, both *C*_max_/MIC and AUC/MIC correlate with efficacy.	^45–48^
*Triterpenoids*				
Ibrexafungerp	AUC_0-24_/MIC	AUC_0-24_/MIC target of 17.4 for *N. glabrata*; *C*_min_ >0.5 mg/L for efficacy	Oral bioavailability facilitates PK/PD optimization for resistant *Candida* spp., including *Candido. auris*.	^49–51^
*Pyrimidine analogues*				
Flucytosine	%*T* > MIC	>75% *T* > MIC associated with optimal efficacy for *Cryptococcus* spp. and *Candida* spp.	Used exclusively in combination therapy to meet PK/PDTs and prevent resistance.	^3,52^

AUC_0-24_/MIC, area under the curve (0-24 h) to minimum inhibitory concentration; *Candido. auris*, *Candidozyma auris*; Cl, clearance; *C*_max_/MIC, maximum concentration to minimum inhibitory concentration; *C*_min_, minimum concentration; fAUC_0-168_/MIC, free area under the concentration–time curve (0–168 h) to minimum inhibitory concentration; %*T* > MIC, percentage of time above minimum inhibitory concentration; *C. albicans*, *Candida albicans*; PK/PD, pharmacokinetic/pharmacodynamic; *N. glabrata*, *Nakaseomyces glabrata*; TDM, therapeutic drug monitoring; *t*_1/2_, half-life.

^a^No validated *Candido. auris*–specific AUC_0-24_/MIC targets have been established for any azole agent in formal PK/PD studies.

^b^In an *in vitro* PK/PD model for *Candido. auris*, the liposomal amphotericin B *C*_max_/MIC target associated with stasis was ∼9; doses of 3 and 5 mg/kg achieved >95% probability of target attainment for MICs ≤1 and 2 mg/L, respectively.^41^

^c^Rezafungin fAUC_0-24_/MIC targets for *Candido. auris* were 1.88 for net stasis and 5.77 for 1-log kill in a neutropenic murine IC model.^47^

The PDT is distinct from PK/PD indices. It represents a numerical threshold, indicating the minimum drug exposure needed to achieve a specific therapeutic outcome.^22,53^ A PDT might specify the minimum AUC/MIC required for a 90% reduction in pathogen load. Achieving an adequate PDT is crucial, as knowledge of the MIC alone is insufficient to ensure treatment success. The MIC must be considered alongside drug exposure to confirm that the relevant PK/PDT has been met.^22,53^ The more drug exposure exceeds the relevant PK/PDT in relation to the organism’s MIC, the greater the likelihood of treatment success.^22^

Pathogens with MICs that exceed achievable drug concentrations result in inadequate PK/PD indices, allowing residual pathogen growth and treatment failure, and may favour selection of less-susceptible subpopulations.^53^ This occurs because low or fluctuating drug concentrations only partially suppress susceptible organisms, whilst permitting less-susceptible subpopulations, which may carry fitness costs but gain a selective advantage under drug pressure, to replicate and expand.^58^ Importantly, a high MIC may indicate that resistance is already present in the infecting organism, reflecting inherently reduced susceptibility; in such cases, achievable drug concentrations are unlikely to meet the PDT and treatment failure may occur from the outset.

Given the variability in human PKs, several complementary approaches are used to help ensure that dosing achieves PDTs. Population PK modelling characterizes a drug’s typical PK behaviour within a patient population and identifies key covariates, such as body weight, renal function or critical illness, that drive interindividual differences in drug exposure. Monte Carlo simulation builds on this by integrating PK distributions with MIC data or predefined PK/PDTs to estimate the probability of target attainment (PTA) for candidate dosing regimens before treatment is initiated. TDM differs in that it measures actual drug concentrations in the individual patient, enabling real-time dose individualization.

In practice, these methods are complementary: population modelling and Monte Carlo simulation inform initial dose selection and support validation of MIC breakpoints and can help define dosing frameworks for specific patient groups, whereas TDM refines dosing at the bedside in the individual patient. From a stewardship perspective, these approaches also support ‘right drug, right dose, right time’ prescribing by reducing avoidable underexposure, unnecessary prolonged therapy and poorly targeted antifungal use. Resistance becomes a concern when PTA declines sharply at higher MICs, when achievable drug exposures cannot meet the relevant PK/PDT or when persistently subtherapeutic exposure favours selection of less-susceptible subpopulations.^59,60^ For example, in critically ill patients, where altered volume of distribution and clearance can undermine standard dosing, Monte Carlo simulation can optimize intermittent caspofungin regimens.^61^ Similarly, when PTA collapses against isolates with elevated MICs, this may indicate reduced susceptibility and heightened risk of treatment failure,^60,62^ warranting confirmatory susceptibility testing and, where appropriate, a change in therapy.

### MSW considerations

The MSW is the range of drug concentrations between the MIC and the mutant prevention concentration (MPC; Figure 2). The MPC represents the drug concentration above the MIC that prevents the growth of resistant mutant subpopulations, thereby defining the upper limit of the MSW. Prolonged exposure to drug concentrations within this range can selectively enrich resistant mutants.^63^ This highlights the importance of designing dosing regimens that not only exceed the MIC but, ideally, remain above the MPC to minimize the emergence of resistance.^63^ While the MIC defines the lower boundary of the MSW, maintaining drug concentrations above the MPC is essential to effectively suppress selection of resistant populations. At the same time, in clinical practice, drug concentrations below the MIC will fail to treat the susceptible infection and may, where prolonged, favour selection of resistant subpopulations. Supporting this concept, a study examining the MPC, MSW and mutation frequency for micafungin and anidulafungin in *Nakaseomyces glabrata* revealed resistance emergence at a breakpoint of 1 mg/L.^63^ Resistance was particularly evident at echinocandin concentrations below 2 mg/L, with 21 out of 32 resistant colonies exhibiting *FKS2* mutations.^63^

**Figure 2.**
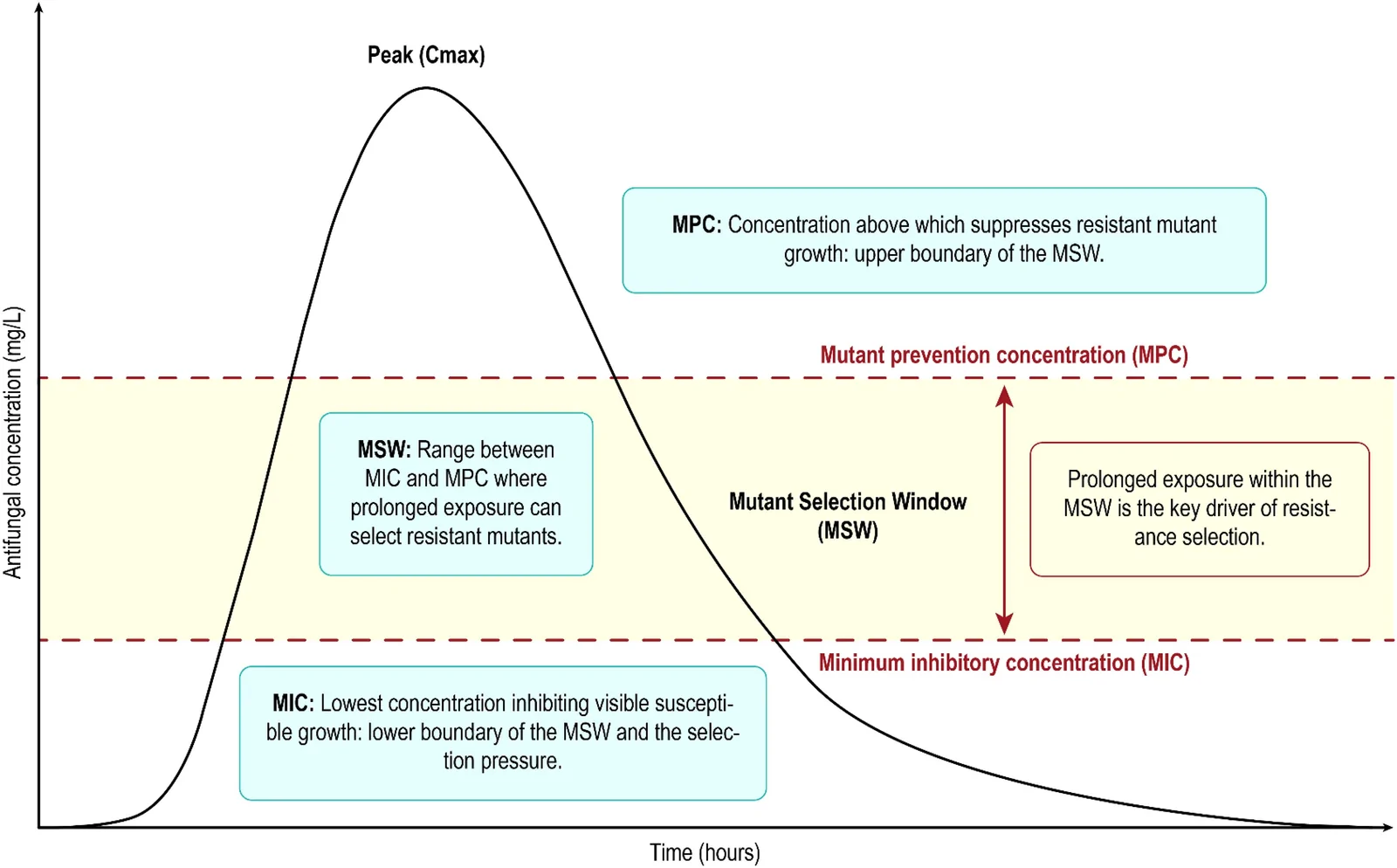
Schematic of the MSW showing how suboptimal or inconsistent antifungal exposure may promote resistance selection. *C*_max_, maximum concentration; MIC, minimum inhibitory concentration; MPC, mutant prevention concentration; MSW, mutant selection window.

## PK/PD drivers in antifungal resistance and strategies for treatment optimization in specific patient groups

### PK variability

Physiological changes in critically ill patients, such as altered hydration, haemodynamics, obesity, tissue perfusion and DDIs, can significantly influence drug distribution (Figure 3).^64^

**Figure 3.**
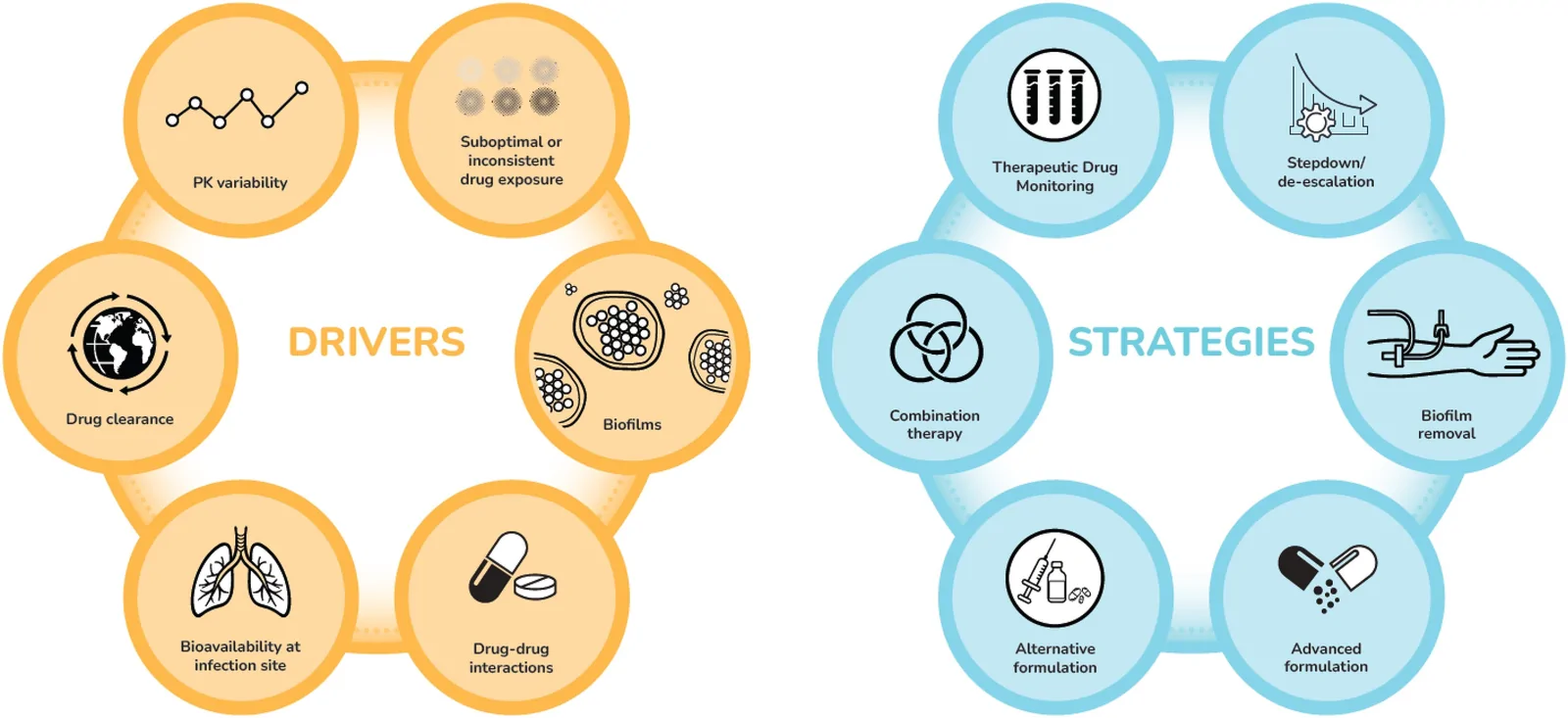
PK/PD strategies for optimizing treatment to combat drivers of antifungal resistance.

Antifungal agents like voriconazole are particularly susceptible to significant inter- and intra-patient PK variability, which is notably heightened when administered orally due to fluctuating absorption rates influenced by gastrointestinal and systemic conditions (Figure 4a).^67^ This variability has direct implications for antifungal resistance. Subtherapeutic levels limit treatment efficacy and expose fungal pathogens to drug concentrations that fall within a range that may permit survival of less-susceptible subpopulations and, where such conditions persist, may contribute to selection of less-susceptible subpopulations.^59^ Repeated or prolonged exposure to such concentrations may apply selective pressure favouring less-susceptible or resistant subpopulations, increasing the risk of treatment failure.^7,60^ Maintaining adequate exposure is therefore not only essential for clinical cure but also a key strategy for suppressing the development of resistance.

**Figure 4.**
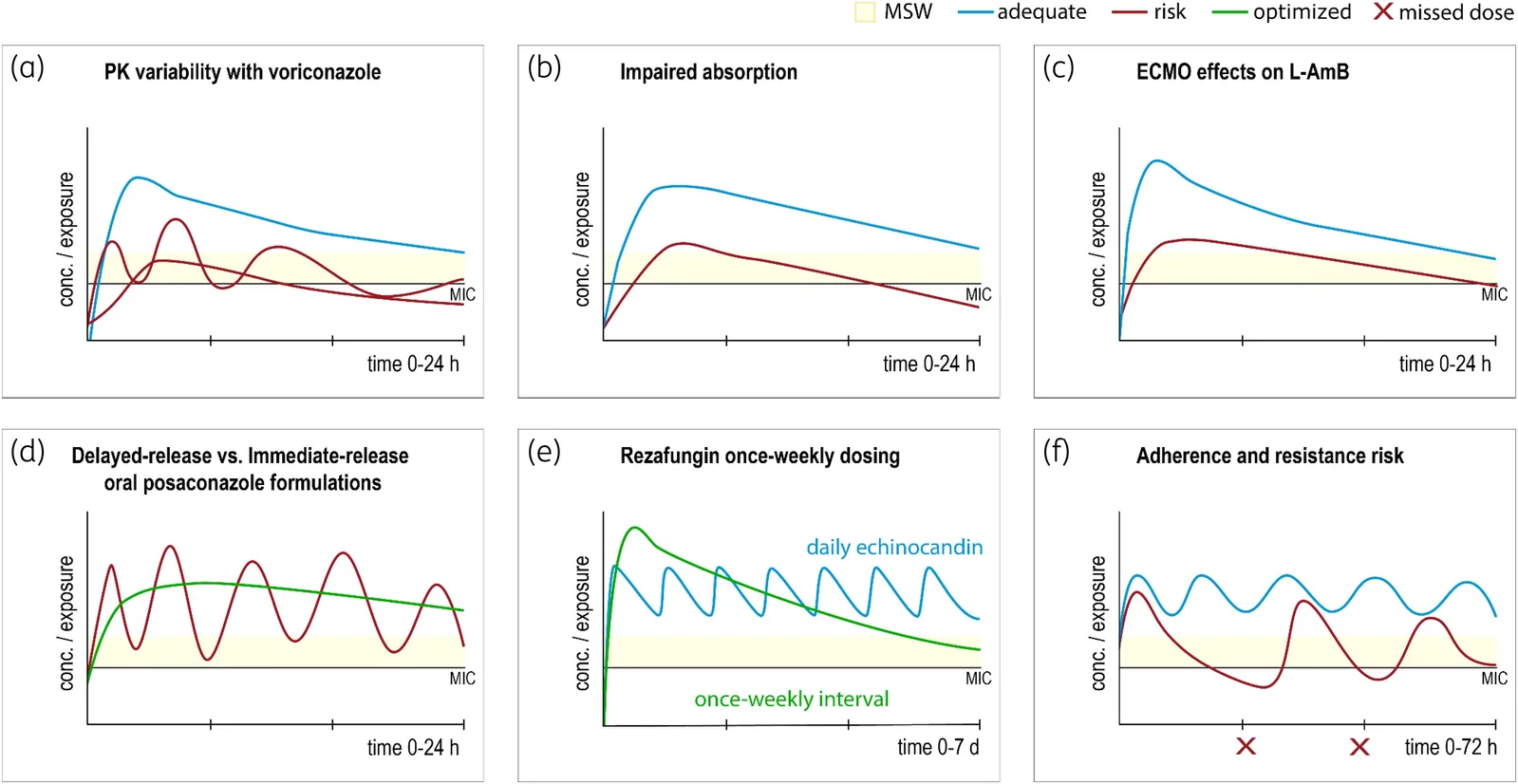
Conceptual antifungal exposure profiles and resistance risk in critically ill patients. Curve heights are illustrative and not scaled to a specific assay or dose. (a) PK variability with voriconazole due to altered haemodynamics, obesity, tissue perfusion, etc., creating periods of subtherapeutic exposure that may select resistant fungal subpopulations. (b) Impaired absorption lowers antifungal exposure and prolongs time within the MSW, supporting the need for TDM and strategies to minimize interacting factors. (c) ECMO may reduce effective L-AmB exposure through circuit sequestration and altered volume of distribution, leading to subtherapeutic concentrations and reduced efficacy. (d) Delayed-release posaconazole achieves steadier plasma concentrations than earlier formulations, helping overcome absorption limitations and maintain target exposure. (e) Rezafungin produces high front-loaded exposure and sustained concentrations above typical MIC values, enabling once-weekly dosing without compromising efficacy.^45,65,66^ In contrast, daily echinocandins produce more frequent peak–trough fluctuations, with concentrations that may approach lower exposure ranges between doses depending on the agent and clinical context. (f) Missed or irregular dosing results in fluctuating antifungal concentrations that repeatedly fall within the MSW, increasing the likelihood of selection of resistant subpopulations. conc., concentration; ECMO, extracorporeal membrane oxygenation; L-AmB, liposomal amphotericin B; MIC, minimum inhibitory concentration; MSW, mutant selection window; PK, pharmacokinetic; TDM, therapeutic drug monitoring.

TDM is essential for most azoles due to their narrow therapeutic index, variability in absorption and metabolism and their tendency for limited distribution into certain tissues.^68^ Achieving effective drug levels at sites like the central nervous system (CNS) or ocular tissues can be challenging, as poor penetration may limit efficacy.^69^ DDIs can further undermine azole exposure. Proton pump inhibitors (PPIs) raise gastric pH and reduce the dissolution and absorption of pH-dependent azoles, with itraconazole capsules being especially affected, leading to lower and more erratic plasma concentrations.^70,71^ These pH-related effects on itraconazole dissolution and absorption further add to the variability already associated with food intake and formulation differences.^72^ Reduced absorption may push drug exposure into a range that promotes resistance selection (Figure 4b), underscoring the importance of TDM and, where possible, the use of formulations or co-medication strategies that minimize these interactions.^27,68^ Conversely, fluconazole stands out as the only antifungal therapy reliably excreted in the urine at concentrations that exceed the MIC, even for organisms that would be classified as resistant by standard breakpoints. Because such breakpoints are not specific to the high drug concentrations achievable in urine, this makes fluconazole a potential treatment option for azole-resistant candiduria, where high urinary drug concentrations can still be therapeutically effective.^73^

While azoles demand frequent monitoring, antifungal treatments with more stable PK profiles, such as liposomal amphotericin B (L-AmB), generally do not require routine TDM. However, important exceptions exist, particularly in critically ill patients, including those receiving ECMO. ECMO can drastically alter the PK of antifungals, including L-AmB, through drug sequestration within circuit components and an increased volume of distribution, potentially resulting in subtherapeutic plasma concentrations and reduced efficacy (Figure 4c).^74,75^ In addition, physiological changes during critical illness, such as increased capillary permeability or fluid shifts during the acute phase, can significantly alter the drug’s PK properties.^64,76^ These changes may increase the volume of distribution, lower plasma concentrations and reduce treatment effectiveness.

In contrast, delayed-release formulations of posaconazole provide more steady plasma concentrations and help overcome the absorption limitations associated with earlier release formulations (Figure 4d).^77^ Similarly, rezafungin differs from other echinocandins in that its once-weekly dosing may simplify the management of invasive candidiasis (IC) and may improve treatment adherence, owing to its high front-loaded exposure and sustained concentrations above typical MIC values (Figure 4e).^78^ However, evidence that these PK features improve adherence, prevent relapse or reduce resistance in routine practice remains limited to date. More broadly, this section highlights that practical strategies such as TDM, formulation selection, management of interacting co-medications and dose individualization in critical illness are central to reducing exposure variability and improving target attainment.

### Suboptimal or inconsistent drug exposure

Poor or inconsistent adherence to treatment is a major contributor to suboptimal exposure, since missed or irregular dosing produces fluctuating plasma concentrations that repeatedly fall within the MSW. This creates opportunities for resistant subpopulations to emerge, particularly during prolonged courses of oral antifungal therapy (Figure 4f). Alongside adherence, exposure to orally administered azoles may also be reduced by impaired absorption (e.g. compromised drug absorption due to cancer treatment), contributing to variability in drug concentrations and potentially compromising treatment efficacy.^64^ Addressing these challenges may require alternative administration routes or dose adjustments to ensure effective therapeutic levels.^64^ For slow-release formulations of posaconazole, absorption can be enhanced by a lipid-rich diet.^79^ Similarly, itraconazole absorption improves at lower gastric pH, further emphasizing the importance of adherence and of dietary and physiological factors in optimizing antifungal therapy to maximize efficacy and limit resistance.^70,72^

Rezafungin’s PK profile, including prolonged half-life, front-loaded exposure and once-weekly dosing, may help maintain echinocandin exposure in selected clinical situations where prolonged intravenous therapy is required or where consistent daily administration is difficult.^45,65,66^ This has been illustrated in a recent case report of relapsing *Rasamsonia argillacea* pneumonia, in which infection control was achieved after a switch from micafungin to rezafungin.^54^ However, interpretation should be made with caution, as the report describes a single complex case and differences in treatment duration and clinical context preclude any direct comparison between agents. Similarly, rezafungin has been used in an individual case of recurrent *N. glabrata* following prior antifungal therapy.^80^ The clinical outcome was favourable, highlighting the potential relevance of sustained drug exposure in complex infections, which remains to be validated in comparative studies.^80^ Taken together, the most actionable strategies for this problem remain improving adherence, addressing impaired absorption, selecting formulations that support consistent exposure and adapting dose or route of administration when target concentrations are not reliably achieved.

### Drug–drug interactions

Patients with IFDs often have multiple comorbidities and are on various medications, increasing the likelihood of PK DDIs, which can unintentionally result in more severe side effects or a reduction in treatment effectiveness.^64,81,82^ Antifungal agents, particularly azoles, can significantly impact the PK/PD of other therapies. As potent inhibitors of the cytochrome P450 enzymes, azoles like voriconazole can increase the levels of other drugs metabolized by these enzymes, such as warfarin, tacrolimus and venetoclax.^64^ This can lead to an increased risk of bleeding or toxicity, necessitating careful monitoring and dose adjustments of the antifungal agents and/or other medications.^64,83,84^ Importantly, although such DDIs can have clinically significant consequences, they raise antifungal or partner-drug exposure and so are not, in themselves, a recognized mechanism for the development of antifungal resistance.

In contrast, interactions that lower azole exposure carry a distinct and arguably more concerning risk. When co-medications reduce azole plasma concentrations below the therapeutic range, they can compromise antifungal therapy through inadequate exposure, leading not only to treatment failure but also, where prolonged, potentially favouring selection of less-susceptible or resistant strains.^85^ Several mechanisms drive this effect. Strong enzyme inducers such as rifampicin and rifabutin accelerate azole clearance and substantially reduce plasma concentrations.^86^ Similarly, antiepileptic drugs like phenytoin can lower itraconazole levels by over 90%, greatly diminishing efficacy and risking treatment failure.^87^ PPIs may further reduce the absorption of pH-dependent azoles such as itraconazole by raising gastric pH.^71^ Where these interactions push drug exposure below the MIC or into the MSW, the resulting selective pressure can favour less-susceptible subpopulations; consistent with this, studies using TDM have linked subtherapeutic azole levels to the rapid emergence of azole resistance.^88^

Some compounds, including the macrolide antibiotics clarithromycin and erythromycin and the immunosuppressant cyclosporin, can inhibit the metabolic breakdown of some antifungal agents.^64,89^ These DDIs may lead to higher-than-intended antifungal concentrations, thereby increasing the risk of adverse effects. However, they would not generally be considered a direct mechanism of antifungal resistance development.^64,90^

### PK considerations with combined antifungal therapy

Combining antifungal agents is practised to enhance antifungal efficacy by leveraging drugs with different mechanisms of action (e.g. AmB and flucytosine).^64^ However, it is important to highlight that combined antifungal therapy can also result in PK/PD complexities, including potential synergistic or antagonistic interactions, which may contribute to treatment failure or resistance development, and is fully reviewed elsewhere.^91,92^ Antagonism rarely arises, requiring two agents to act on the same target or alter each other’s concentrations. Instead, it more commonly occurs when one agent changes fungal physiology or reduces the availability of the second drug’s target, thereby diminishing its activity. A representative example is the interaction between azoles and AmB. By inhibiting ergosterol biosynthesis, azoles deplete the membrane ergosterol for AmB binding, which can reduce polyene activity.^93,94^ However, because AmB targets the sterol itself rather than a protein, it remains unclear whether this type of antagonism carries direct implications for resistance; importantly, in this context antagonism denotes that the combined effect is less than the summed activities of the two agents, but not necessarily less than the activity of either agent alone, so additive benefit over monotherapy may still occur. A preclinical study examined the interaction between posaconazole and olorofim in a murine model of pulmonary invasive aspergillosis (IA).^95^ Although both drugs were effective individually, their combination showed antagonistic effects, particularly in triazole-resistant strains, where the combination was less effective than olorofim alone.^95^ In *Aspergillus* spp., combining olorofim with mould-active azoles has also been shown to increase olorofim MICs.^96^ Although the elevated olorofim MIC remained within the wild-type distribution, it is unclear whether this shift could still favour selection of resistant subpopulations during combined therapy. Phase II analyses did not identify this as a clear clinical problem, but the interaction warrants continued scrutiny.^97^ The antagonism appears to be unidirectional towards olorofim and is likely related to azole-induced upregulation of the pyrimidine biosynthesis pathway.^98^

Similarly, an *in vitro* study investigating a triple antifungal combination consisting of caspofungin, voriconazole and AmB against *Aspergillus* spp. reported concentration-dependent interactions.^99^ Synergistic effects were observed at lower concentrations of AmB (0.10–0.22 mg/L) and voriconazole (0.07–0.15 mg/L) alongside caspofungin [fractional inhibitory concentration (FIC) 0.49–0.57], suggesting that combination therapy at these levels may enhance fungicidal activity and help suppress the emergence of resistance in less-susceptible isolates. In contrast, at higher AmB (0.3–0.5 mg/L) and voriconazole concentrations (0.23–0.68 mg/L), antagonistic effects appeared (FIC 1.65–2.15), which could diminish therapeutic efficacy and potentially complicate the treatment of resistant infections.^99^

### Inadequate penetration at the infection site

Effective treatment of IFDs relies on antifungal drugs reaching the infection site in adequate concentrations, although penetration can be inconsistent and presents a significant challenge.^64^ For airway diseases, systemic antifungal therapies often fail to adequately traverse the airways and exit the pulmonary parenchyma, emphasizing the need for inhaled antifungal agents.^100^ Novel nebulized formulations, such as opelconazole, offer a targeted approach, delivering high drug concentrations directly to the site of infection while minimizing systemic exposure. This makes them ideal for treating airway disease, but potentially less so if the infection become invasive in nature and migrates into the lung parenchyma.^101^ The wide clinical spectrum and fluidity of diseases like aspergillosis must be considered when selecting therapy, both in terms of efficacy and the potential for resistance selection or development, particularly because antifungal levels may vary across anatomical sites.

Such variability is shaped heavily by the chemical properties of individual agents. Posaconazole’s lipophilicity allows it to linger in cell membranes and extend its half-life, thereby sustaining drug exposure at the site of infection and potentially limiting the selection pressure that can drive resistance in less-susceptible isolates. In contrast, the more hydrophilic voriconazole is primarily distributed within the circulation, resulting in a different PK profile that may yield more variable tissue concentrations and, where exposure falls below effective thresholds, could favour the emergence of resistant subpopulations.^102^

The chemical and PK properties that determine where an antifungal accumulates, and at what concentration, vary considerably between agents, and these differences carry direct implications for resistance (Table 2). Because antifungal levels can vary between anatomical sites, a drug may achieve adequate concentrations at one infection focus yet remain subtherapeutic at another, exposing less susceptible subpopulations to selection pressure. By summarizing how differences in tissue distribution, protein binding, half-life, elimination, intracellular uptake and site-specific penetration shape antifungal exposure, Table 2 provides the PK context for resistance, illustrating how variation in achievable concentrations at the site of infection can influence selection pressure and contribute to treatment failure even when an organism remains susceptible *in vitro*.

**Table 2.** Comparative tissue distribution and PK features of current antifungal agents^56,103–105^

Agent	Volume of distribution (L/kg)	Half-life	Protein binding	CNS/CSF penetration	Lung tissue/ELF	Ocular penetration	Urine conc.	Intracellular uptake	Tissue accumulation
*Triazoles*									
Fluconazole	Moderate (∼0.7)	Long (∼30 h)	Low (10%)	High (0.5–0.9 CSF; up to 2.4 tissue)	High (∼1.1–2.4 tissue; ELF ∼0.5–5)	Moderate/high (∼0.8 VH/AH)	High (active renal excretion; urine exceeds plasma)	Low–moderate	Moderate (saliva, sputum, urine, peritoneal fluid)
Itraconazole	High (∼11)	Long (∼20 h)	Very high (99.8%)	Low (0–0.12; negligible)	Moderate/high (0.9–7 tissue; ELF ∼0.3)	Low (∼0.28 VH; ∼0.65 AH)	Negligible	Moderate	High (skin, nails, adipose)
Voriconazole	Moderate–high (∼4.6)	Variable (6–12 h, nonlinear)	Moderate (∼58%)	High (0.2–1.0 CSF; ∼1–2 tissue)	High (1.1–7.4 tissue; ELF ∼6–12.5)	Moderate–high (∼0.38 VH; ∼0.51 AH)	Low (metabolites predominate; <2% unchanged)	Moderate	Moderate (brain, lung, liver)
Posaconazole	High (∼10–20)	Long (15–35 h)	Very high (∼99%)	Low–moderate (<0.5 to 0.5–5; variable, often low)	High (ELF 0.5–5; tissue >5)	Low (<0.5 VH; ∼0.26 AH)	Negligible (minimal parent in urine)	High	High (lipid-rich tissues: adipose, liver, spleen, lung)
Isavuconazole	High (∼6.5)	Very long (80–130 h)	Very high (>99%)	High (∼1.5–1.8 tissue)	High (2–6 tissue)	Low–moderate (limited data)	Low unchanged parent drug in urine	Moderate	High (lung, liver, adipose, kidney)
*Echinocandins*									
Anidulafungin	Low (0.38–0.63)	Long (∼40–50 h)	High (∼84%)	Low (<0.5)	Low–moderate (ELF <0.5; tissue ∼10)	Low (<0.5 VH; <0.5 AH)	Negligible (biliary/faecal elimination; no renal excretion)	Low	Moderate (liver, lung)
Caspofungin	Low (∼0.12)	Intermediate (∼9–11 h, β-phase)	Very high (∼97%)	Low (<0.5)	Low (ELF not well defined; tissue <0.5)	Low (<0.5 VH; <0.5 AH)	Low/negligible (minimal unchanged)	Low–moderate	Moderate–high (liver predominant; slow tissue distribution)
Micafungin	Low (0.39)	Intermediate (∼14–17 h)	Very high (∼99%)	Low (<0.5)	Low–moderate (ELF <0.5; tissue 0.5–5)	Low (<0.5 VH; <0.5 AH)	Negligible (biliary elimination predominates)	Low–moderate	High (liver, spleen, lung)
Rezafungin	Low (0.36–0.54; dose-dependent)	Very long (∼130–150 h)	High (ND)	Low (∼0.6)	High (∼4.1–4.6)	ND	Low/negligible (biliary elimination predominates)	Low (ND; not well characterized)	High (kidney, liver, lung, spleen; preclinical data)
Ibrexafungerp	Moderate (4.4–5.3)	Long (∼20–30 h)	High (ND)	Low (0–0.11)	High (∼26)	High (uvea only) (∼117; AH/VH not measured)	Low	Low (ND; not well characterized)	High (lung, spleen, liver)
*Polyenes*									
Amphotericin B (conventional)	Moderate (1.8–2.3)	Long (∼24 h; terminal phase up to several days)	Very high (>95%)	Low (<0.5–0.5)	Moderate (ELF 0.5–5; tissue 0.5–5)	Low (<0.5 VH; <0.5 AH)	Low (low urinary recovery of active drug)	High (phagocyte/macrophage uptake)	High (liver, spleen, lung, kidney; prolonged retention)
Liposomal amphotericin B	High (4.6–7.9)	Long (∼7–10 h; prolonged terminal phase)	Very high (>95%)	Low (<0.5)	Moderate (ELF 0.5–5; tissue 0.5–5)	Low–moderate (<0.5–5 VH/AH)	Low	High (RES/macrophage uptake)	Very high (liver, spleen, lung; reduced kidney accumulation versus conventional)
*Antimetabolite*									
5-FC	Moderate (∼0.7)	Short (∼3–6 h)	Low (minimal; <5%)	High (∼0.7–1)	High (good lung tissue penetration; ELF approaches plasma)	Moderate (penetrates ocular tissues)	High (predominantly unchanged renal excretion; ∼90%)	Low	Low

Penetration values represent the ratio of serum or plasma concentration to tissue concentration, grouped into three categories (<0.5, 0.5–5 and >5) or presented as exact values where reported in the literature. These categories are consistent with the 2014 classifications from Felton *et al*.,^56^ and exact values are included where available to reflect the most up-to-date information.

5-FC, flucytosine; AH, aqueous humour; CNS, central nervous system; CSF, cerebrospinal fluid; ELF, epithelial lining fluid; ND, not determined; RES, reticuloendothelial system; VH, vitreous humour.

Penetration challenges are not limited to airway diseases but extend to other difficult-to-treat foci, such as CNS infections, eye infections and biofilms, where certain systemic antifungal therapies may not attain efficacious levels, which is both a treatment and resistance concern.^106^ Fungal endophthalmitis remains a therapeutic challenge due to the need for drugs to effectively traverse multiple ocular barriers.^107^ Intrathecal administration offers an alternative for addressing CNS infections when systemic drug delivery proves inadequate. AmB, for example, poorly penetrates the cerebrospinal fluid (CSF) under normal conditions.^64^ This limitation can be partially overcome by combining AmB with flucytosine, which penetrates the CSF efficiently and is particularly effective for cryptococcal meningitis.^64^ Interestingly, inflammation of the blood–brain barrier facilitates the penetration of antifungal agents and antibiotics, enhancing outcomes for CNS infections.^108^

Echinocandins, although effective systemic antifungals, exhibit poor penetration into certain tissues compared to azoles.^56^ Azoles not only provide better penetration but are also the preferred treatment for respiratory aspergillosis due to their proven efficacy.^109^ However, whether azoles act in a fungistatic or fungicidal manner varies between fungal species. Against organisms for which their activity is fungistatic, their effectiveness may be limited in severe infections compared with fungicidal agents and suboptimal drug levels may promote resistance. For example, a step-down from an echinocandin (fungicidal to yeast) to fluconazole (fungistatic to yeast) can sometimes result in suboptimal outcomes, especially in severe or refractory yeast infections.^110^ Isavuconazole offers potential benefits such as once-daily dosing, broad-spectrum activity and reduced nephrotoxicity, but its limitations include variable efficacy in azole-resistant strains and the need for TDM in complex cases.^111,112^

Clinical evidence underscores variability in achieving therapeutic levels. A prospective study of hospitalized patients revealed that only 64.3% (54/84) achieved adequate serum antifungal levels despite proper dosing of various antifungal drugs, including micafungin, fluconazole, voriconazole, anidulafungin, posaconazole and caspofungin.^113^ Importantly, attainment of adequate serum antifungal concentrations was associated with better clinical outcomes: favourable outcomes occurred in 96.3% (52/54) of patients who achieved target levels compared with 83.3% (25/30) of those who did not, while poor outcomes occurred in 3.7% versus 16.7%, respectively. For example, one patient receiving micafungin prophylaxis developed persistent candidaemia due to subtherapeutic serum concentrations; dose adjustment guided by serum monitoring resolved the infection. This case illustrates treatment failure due to inadequate exposure rather than acquired resistance and highlights the need for individualized dosing of intravenous agents in some patients.^113^

Penetration challenges are also evident in pulmonary infections. AmB (regardless of formulation) achieves moderate pulmonary exposure, with concentrations in both epithelial lining fluid (ELF) and lung tissue generally ranging from 0.5 to 5 mg/L;^103^ however, ELF concentrations are typically lower than total lung tissue concentrations and are often reported in the range of 0.4–1.6 mg/L.^114^ Standard doses of L-AmB often result in ELF concentrations at or below the MICs for key fungal pathogens,^114^ potentially placing exposure within the MSW and thereby favouring selection of less-susceptible subpopulations. This underscores the critical need to ensure sufficient drug levels at infection sites to treat invasive pulmonary mycoses effectively, with PK/PD frameworks guiding the optimization of drug concentrations at target sites.^114^

Lipid formulations address several of these limitations: L-AmB can be administered at higher doses than conventional AmB, improving plasma exposure and penetration into the lungs and CNS while reducing nephrotoxicity and its long half-life and tissue retention allow single or intermittent dosing regimens without diminishing antifungal efficacy.^40,115,116^ Clinical trials have demonstrated L-AmB’s superior safety profile, with fewer infusion reactions and reduced renal toxicity, making it an option for invasive mycoses like aspergillosis, mucormycosis and candidaemia.^40,115^

Notably, new manufacturers have introduced generic L-AmB products, expanding access and affordability.^117^ This formulation is widely preferred over conventional AmB for treatments like aspergillosis and candidaemia, with higher recommended dosages ranging from 1 to 6 mg/kg/day.^40^

Ibrexafungerp, a novel oral glucan synthase inhibitor, may help address some penetration-related challenges because it achieves high tissue concentrations, including in biofilm-associated infections.^118^ Although it inhibits β-D-glucan synthesis like the echinocandins, its distinct binding interactions confer limited cross-resistance, allowing it to retain activity against many echinocandin-resistant strains.^118^ Reports of use in refractory cases of IC or pulmonary aspergillosis remain limited and should be interpreted cautiously; at present, it is licenced only for the treatment of superficial candidiasis.^119^ In practice, penetration-related challenges may be addressed by selecting agents with more favourable site-specific distribution, using alternative formulations or routes of delivery where appropriate and integrating PK/PD considerations into drug and dose selection for the anatomical focus of infection.

### Biofilms

As reviewed by Ramage *et al.*, fungal biofilms are increasingly recognized as a critical barrier to effective antifungal therapy, contributing significantly to the persistence and complexity of fungal infections.^120^ Frequently found on medical devices or tissues, fungal biofilms resist antifungal treatment and eradication often requires not only higher drug concentrations but a shift to alternative agents whose activity is unaffected by the biofilm lifestyle.^121^ This elevated requirement reflects reduced susceptibility and, where therapeutic concentrations cannot be sustained *in vivo*, risks subtherapeutic exposure that may select for resistant subpopulations. The extracellular matrix produced by the fungal cells within the biofilm provides a protective barrier. However, small fragments of biofilm exhibit a high level of tolerance in the absence of penetration issues, which indicates that the persistence is due to the biofilm lifestyle itself, namely the unique metabolic and physiological states of biofilm-associated cells.^120^ Metabolic dormancy combined with efflux pump upregulation make biofilm-associated infections notoriously difficult to treat.^120^ Mixed-species biofilms, such as *Candida albicans* adhering to *Streptococcus* spp. in the oral cavity, further complicate treatment by enhancing antifungal resistance through synergistic microbial interactions, potentially including increased matrix production and altered biofilm architecture that limit drug penetration, as well as interspecies signalling that promote persistence.^122,123^

The biofilm lifestyle particularly affects the activity of azole antifungals: although these agents may be highly active against planktonic yeast cells, this activity is largely lost against yeast biofilms.^120,123–125^ In contrast, echinocandins and lipid polyenes have good activity against biofilm-forming yeasts as they target essential cell wall and membrane components, overcoming the extracellular matrix and metabolic adaptations.^126–128^ Emerging strategies to combat *Candida* biofilms include biofilm-disrupting agents, novel antifungal therapies and combination treatments.^120^ Novel agents like ibrexafungerp, a first-in-class oral triterpenoid targeting the β-glucan synthase pathway, have demonstrated anti-biofilm activity against multiple *Candida* spp., including *C. albicans*.^124^ Similarly, rezafungin has demonstrated non-inferiority to caspofungin in clinical trials for candidaemia/IC and has shown anti-biofilm activity in preclinical and early clinical reports, particularly against pan-fungal resistant pathogens like *Candido. auris*.^124,129,130^ Some drug combinations, such as AmB with echinocandins also show promise. Animal studies indicate that combining systemic and lock therapies with micafungin effectively eradicates catheter-based biofilms and infections caused by *C. albicans* or *Candida parapsilosis*, particularly in neutropenic conditions, offering clinical benefits when catheter removal is not feasible.^131^ Agents that reliably achieve the high local concentrations required for biofilm eradication, together with device management where feasible and selection of agents with retained anti-biofilm activity, may also reduce the risk of selecting for resistant organisms.

### Drug clearance

The clearance of antifungal drugs can be significantly affected by the patient’s renal and hepatic function, and these variations carry direct implications for the emergence of resistance.^64^ When clearance shifts drug exposure away from therapeutic targets, the result is either toxic accumulation or, more concerning from a resistance standpoint, subtherapeutic concentrations that permit fungal survival and adaptation. For example, fluconazole is eliminated primarily unmetabolized through the kidneys, so patients with renal impairment may require dose adjustments to prevent accumulation and toxicity.^64^ Conversely, unrecognized changes in renal elimination can leave concentrations too low to fully suppress the pathogen, creating a window in which less-susceptible subpopulations can persist and expand. Voriconazole, which is metabolized by the liver, requires careful dosing in patients with hepatic impairment or with interacting medications; accelerated hepatic clearance is especially problematic because it can drop drug levels below the threshold needed to clear the infection, resulting in inadequate exposure and possible clinical failure, and, where such low-level exposure persists, potentially favouring selection of less-susceptible isolates.^64^ AmB is cleared primarily by the kidneys and reduced renal function can lead to drug accumulation and increased risk of nephrotoxicity, necessitating close monitoring and potential dose adjustments.^132,133^ On the other hand, most echinocandins, with the exception of rezafungin, are metabolized primarily by the liver and excreted through faeces, making hepatic function a key determinant for their clearance and sometimes necessitating dose adjustment in patients with liver dysfunction; however, they are generally considered ‘clean drugs’, in the sense that they have a low propensity for clinically significant DDIs and generally predictable PKs.^134^

Genetic variation in metabolism adds a further layer of resistance risk. Some patients exhibit enhanced metabolism of azoles due to genetic mutations, particularly in the CYP2C19 enzyme.^135^ Variants like CYP2C19*17 result in rapid or ultra-rapid metabolism, leading to lower drug concentrations and reduced efficacy of treatments like voriconazole.^135,136^ These mutations significantly hamper the efficacy of azole treatments, because affected individuals clear the drug too quickly for it to reach and maintain therapeutic concentrations. The consequence is not only treatment failure but also a sustained period of low-level drug exposure, which represents inadequate exposure and, where prolonged, may exert selective pressure favouring less-susceptible subpopulations. This rapid clearance can be exacerbated by DDIs that activate liver enzymes or conditions that promote enhanced excretion, both of which deepen the gap between administered dose and effective tissue exposure.^64^ Recognizing rapid metabolizer status and adjusting the dose accordingly or selecting an alternative agent, can help restore adequate exposure and limit this resistance-promoting window.

Excretory capacity poses related challenges. AmB, especially in its conventional formulation, carries a significant risk of nephrotoxicity, and in patients with renal impairment it may accumulate to toxic levels unless dosing is carefully adjusted or lipid-based formulations are used.^137,138^ Yet, overly conservative dosing can leave concentrations subtherapeutic, promoting persistent infection and the selection of resistant organisms. Lipid-based formulations resolve this tension, sustaining effective exposure while reducing renal injury.

Echinocandins, while generally well tolerated, are primarily metabolized through non-enzymatic degradation and hepatic pathways, making them less dependent on renal clearance.^139^ This clearance profile reduces the risk of nephrotoxicity, even in patients with renal impairment, and makes echinocandins a safer option for such individuals. However, dose adjustments may still be necessary in cases of severe hepatic dysfunction, obesity or in patients undergoing ECMO, as these conditions can alter drug clearance, leading to drug accumulation and potential toxicity.^140^ Conversely, altered clearance or distribution in these settings can also leave exposure subtherapeutic, reducing efficacy and favouring the persistence of less-susceptible organisms.

## Limitations, research gaps and future directions

Several limitations of this review should be acknowledged. First, this is a narrative rather than a systematic review, and evidence was prioritized pragmatically. As a result, our selection of studies may reflect a degree of publication bias, since positive or novel findings are more likely to appear in the literature and be cited. Second, for several questions, particularly those concerning tissue penetration and the relationship between drug exposure and resistance, the strongest available evidence comes from *in vitro* models and animal PK/PD studies. While these data are informative, their translation to the clinical setting is not always direct, and species differences, protein binding and infection-site conditions may all influence real-world outcomes. Third, the evidence base for newer antifungals, including rezafungin, ibrexafungerp and olorofim, remains limited. Randomized controlled trials for these agents are few, and much of the current understanding rests on early-phase studies, modelling analyses, preclinical work and, in some settings, isolated case reports. The dosing and monitoring guidance offered here for these drugs should therefore be regarded as provisional and likely to evolve as further clinical data emerge. In particular, although these approaches are biologically and pharmacologically plausible means of reducing resistance selection pressure, direct clinical evidence demonstrating prevention of acquired resistance remains limited.

Several priorities stand out for future work. Prospective studies of TDM in special populations, including critically ill, obese and organ-impaired patients, are needed to define target exposures and confirm that dose individualization improves outcomes. There is a clear need for better-defined PK/PD breakpoints, supported by data linking exposure to both efficacy and the suppression of resistance. Coordinated, real-world resistance surveillance would help clinicians anticipate shifting susceptibility patterns and adjust empirical strategies accordingly. Finally, robust clinical outcome data for newer agents are essential to establish their place in therapy and to move current dosing recommendations from a largely preclinical footing to firm, evidence-based practice. Addressing these gaps will be central to optimizing antifungal therapy, limiting resistance and reducing treatment failure in IFDs.

### Conclusions

The management of IFDs requires a multifaceted approach that integrates PK/PD optimization, novel antifungal agents and robust stewardship practices, including timely initiation, appropriate drug selection, dose and duration optimization, TDM-guided adjustment where needed and de-escalation or discontinuation when appropriate. Emerging therapies like rezafungin and ibrexafungerp may help address some current treatment limitations, although their precise role in overcoming resistance and biofilm-associated challenges requires further clinical validation, while TDM and advanced modelling techniques can support personalized treatment strategies, particularly through patient-group-informed dosing and individual dose refinement. Addressing PK variability, drug interactions, tissue penetration barriers and impact of fungal biofilms through measures such as TDM, dose individualization, formulation or route selection and stewardship-informed antifungal choice is critical to optimizing exposure and achieving therapeutic success. By leveraging these advancements, clinicians may reduce treatment failure and potentially lessen resistance selection pressure, although direct clinical evidence for prevention of acquired resistance remains limited.

## Transparency declarations

N.V.R. has previously received speaking fees from Mundipharma. P.L.W. has performed diagnostic evaluations and received meeting sponsorship from Associates of Cape Cod, Bruker, Dynamiker and Launch Diagnostics; speaker fees, expert advice fees and meeting sponsorship from Gilead and Mundipharma Research Limited; speaker and expert advice fees from Pfizer and expert advice fees from F2G. R.R.-R. has received speaker fees from Astellas, Basilea, F2G, Gilead Sciences, NAPP/Mundipharma and Pfizer and Scynexis; and been a principal investigator for clinical trials for F2G and Scynexis.
